# Nod2: The intestinal gate keeper

**DOI:** 10.1371/journal.ppat.1006177

**Published:** 2017-03-02

**Authors:** Ziad Al Nabhani, Gilles Dietrich, Jean-Pierre Hugot, Frederick Barreau

**Affiliations:** 1 Laboratoire Inflamex, Université Paris-Diderot Sorbonne Paris-Cité, Paris, France; 2 INSERM, UMR 1149, Paris, France; 3 IRSD, Université de Toulouse, INSERM, INRA, ENVT, UPS, Toulouse, France; 4 Assistance Publique Hôpitaux de Paris, Hôpital Robert Debré, Paris, France; Stony Brook University, UNITED STATES

## Abstract

Nucleotide-binding oligomerization domain 2 (NOD2) is an intracellular pattern recognition receptor that senses bacterial peptidoglycan (PGN)-conserved motifs in cytosol and stimulates host immune response. The association of *NOD2* mutations with a number of inflammatory pathologies, including Crohn disease (CD), Graft-versus-host disease (GVHD), and Blau syndrome, highlights its pivotal role in host–pathogen interactions and inflammatory response. Stimulation of NOD2 by its ligand (muramyl dipeptide) activates pro-inflammatory pathways such as nuclear factor-κB (NF-κB), mitogen-activated protein kinases (MAPKs), and Caspase-1. A loss of NOD2 function may result in a failure in the control of microbial infection, thereby initiating systemic responses and aberrant inflammation. Because the ligand of Nod2 is conserved in both gram-positive and gram-negative bacteria, NOD2 detects a wide variety of microorganisms. Furthermore, current literature evidences that NOD2 is also able to control viruses’ and parasites’ infections. In this review, we present and discuss recent developments about the role of NOD2 in shaping the gut commensal microbiota and pathogens, including bacteria, viruses, and parasites, and the mechanisms by which Nod2 mutations participate in disease occurrence.

## Introduction

The mammalian intestinal tract harbors a community of trillions of bacteria, archaea, fungi, and viruses, which are collectively referred to as the microbiome. It is now well accepted that a mutualistic relationship between host and microbiome is essential for immune homeostasis [1]. The microbiome is required for the development [2] and regulation of intestinal immune responses against commensals and pathogens, thereby maintaining the intestinal homeostasis.

Initiation of the immune response depends on the recognition of microbial-associated molecular patterns (MAMPs) through special cell receptors called pattern recognition receptors (PRRs). PRRs are classified into five distinct genetic and functional clades (for review, see [3]). Most of our knowledge concerning PRRs comes from studies on toll-like receptors (TLRs), which are localized either at the cell surface or within endosomes [4,5]. By contrast, the nucleotide oligomerization domains (Nod)-like receptors (NLRs) are intracellular sensors, including 22 members in humans and 34 members in mice [6]. The activation of multiple PRRs in response to a pathogen triggers nuclear factor-κB (NF-κB), mitogen-activated protein kinases (MAPKs), Caspase-1 activation, and both interleukin 1 (IL-1) and type I interferon (IFN) secretion, inducing inflammation [3].

NOD2, also known as NLRC2, belongs to the NLR family and functions as an intracellular PRR for muramyl dipeptide (MDP) derived from peptidoglycan (PGN) of both gram-positive and gram-negative bacteria [7]. Since its identification in 2001 [8] and its association with Crohn disease (CD) [9,10], the role of NOD2 in both innate and adaptive immune responses gained increasing interest. *NOD2* mutations confer highest risks for CD, but also for Graft-versus-host disease (GVHD) [11] and Blau syndrome [12]. Dysregulation of Nod2 signaling causes or contributes to increased infection risks in human and animal models. This review focuses on the role of NOD2 in the recognition and elimination of commensal and pathogenic bacteria, viruses, and parasites in the gut.

## NOD2 expression, activation, structure, and signaling

In the intestine, NOD2 is expressed by numerous cell types, including hematopoietic cells [13] (such as T cells [14], B cells [15], macrophages [16], dendritic cells [17], and mast cells [18]) and nonhematopoietic cells (such as Paneth cells [19], stem cells [20], goblet cells [21], and enterocytes [22,23]). NOD2 senses MDP, which is derived from partial degradation of PGN [7]. MDP directly binds to the nucleotide-binding domain of NOD2 [24,25] from amino acids 216 to 821 [25] with an optimal efficiency within a pH ranging from 5.0 to 6.5 [24]. NOD2 is able to detect many types of PGN; however, its level of activation is dependent on the PGN’s origin [26]. Following activation, NOD2 activates NF-κB and MAPK signaling [27,28], thereby contributing to host defense via the production of inflammatory cytokines, antimicrobial molecules [29], and mucins [21].

**Fig 1:**
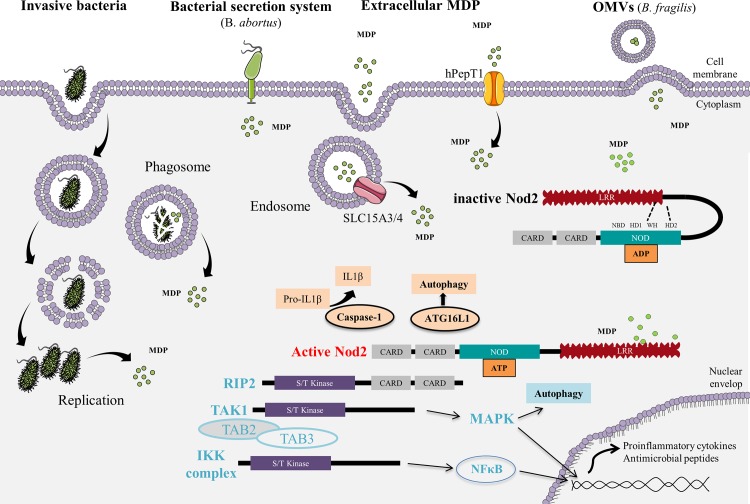
Mechanisms by which MDP enters into cells to trigger Nod2 signaling. Several routes of MDP entry have been evidenced. Host cells can internalize MDP through either phagocytosis of whole bacteria, endocytosis, uptaking of PGN fragments from OMVs, or transmembrane channels such as hPepT1. A new way of Nod2 activation involving the entry of MDP via the apparatus secretion system of bacteria has recently been described. NOD2 activation requires its location to be in the vicinity of the site of MDP delivery. Two peptide transporters (SLC15A3 and SLC15A4) are able to translocate MDP toward the cytosolic compartment. NOD2 protein exhibits three domains, including caspase activation and recruitment domains (CARDs), nucleotide-binding oligomerization domain (NOD), and leucine-rich repeat (LRR). The NOD module contains a nucleotide-binding domain (NBD), a winged helix (WH), and two helix domains (HD1 and HD2). The interaction between NBD and WH, important to stabilize Nod2 in an inactive form, is maintained by adenosine diphosphate (ADP)-mediated packed conformation. Upon ligand binding, HD2 mediates conformational changes of the NBD, WH, and HD1 to allow ADP-ATP exchange, self-oligomerization, and downstream signaling. The effector CARDs mediate intracellular signaling after interaction between the LRR domain and MDP. NOD2 oligomerization induces a signaling complex named nodosome. NOD2 attracts receptor-interacting serine/threonine-protein kinase 2 (RIP2) via a CARD–CARD homotypic interaction, followed by transforming growth factor beta-activated kinase 1 (TAK1) and TAK1 binding proteins 2 and 3 (TAB2 and TAB3). This complex induces the activation of both MAPKs and NF-κB pathways. The interaction of NOD2 with other partners, including Caspase-1 and ATG16L1, results in IL-1β secretion and autophagy, respectively.

The mechanisms by which PGN enters eukaryotic cells and activates NOD2 remain poorly understood, but several routes of entry have been proposed. Host cells can internalize MDP through either phagocytosis of whole bacteria, endocytosis, uptaking of PGN fragments from outer membrane vesicle (OMVs) [30,31], or transmembrane channels such as hPepT1 [32,33]. A new way of Nod2 activation involving the entry of MDP via the apparatus secretion system of bacteria has recently been reported [34]. NOD2 activation requires its location to be in the vicinity of the site of MDP delivery, close to the plasma membrane or endosomes in which two peptide transporters, SLC15A3 and SLC15A4, may transport MDP toward the cytosolic compartment [32] (Fig 1).

*NOD2* protein exhibits three domains, including caspase activation and recruitment domains (CARDs), nucleotide-binding oligomerization domain (NOD), and leucine-rich repeat (LRR). The NOD module contains a nucleotide-binding domain (NBD), a winged helix (WH), and two helix domains (HD1 and HD2). The interaction between NBD and WH, important to stabilize Nod2 in an inactive form, is maintained by ADP-mediated packed conformation [35]. In the absence of MDP binding, the LRR domain prevents NOD2 dimerization. Upon ligand binding, HD2 mediates conformational changes of the NBD, WH, and HD1 to allow ADP-ATP exchange, self-oligomerization, and downstream signaling [36]. The effector CARDs mediate intracellular signaling after interaction between the LRR domain and MDP (Fig 1). NOD2 oligomerization induces a signaling complex named nodosome [37]. The nodosome may be formed at the plasma cell membrane, where bacteria are taken in charge [37]. Among the recruited interactants, NOD2 firstly attracts RIP2 via a CARD–CARD homotypic interaction [8], followed by TAK1 and TAB2 and TAB3 [38]. The kinase activity of TAK1 induces the activation of MAPKs and NF-κB pathways [38]. The interaction of NOD2 with other partners, including Caspase-1 [39] and ATG16L1 [40], results in IL-1β secretion and autophagy, respectively (Fig 1).

## NOD2 and the intestinal microbiota

Humans are colonized by a collection of microbes, the largest numbers of which reside in the distal gut. The human gut contains between 500 and 1,000 bacterial species. There is a gradual increase in bacterial populations all along the small bowel, from approximately 10^4^ colony forming units (CFUs) per gram of luminal content in jejunum to 10^7^ in the ileum, with a preponderance of gram-negative aerobes. By contrast, the human colon is highly colonized with anaerobic bacteria, with about 10^14^ per gram of luminal content. The intestinal microbiota species belong to only eight of the 55 known bacteria phyla (Firmicutes, Bacteroidetes, Actinobacteria, and Proteobacteria phyla being the most widely represented). The gut microbiota acts as a “metabolic organ” through breakdown of indigestible dietary carbohydrates and proteins and generation of fermentation end-products and vitamins. The microbiota contributes also to the intestinal barrier function, which constitutes an obstacle to pathogen invasion of the intestinal mucosa. Commensal bacterial flora is known to be affected by numerous factors, including antibiotics, genetic background, diet, parents, and siblings. Moreover, several human diseases, including inflammatory bowel diseases (IBDs), obesity and metabolic disorders, and infectious and neurological diseases, are linked to a so-called microbiota dysbiosis.

Abnormal interactions between host and microbes (either pathogen or commensal) are involved in IBDs, including CD and ulcerative colitis (UC). IBD physiopathology is associated with significant shifts in the composition of the enteric microbiota (i.e., dysbiosis), notably via an increased richness of the Bacteroidetes, Actinobacteria, and Proteobacteria phyla and a depletion of the Firmicutes phylum [41,42]. The loss of Firmicutes is mostly due to the reduction of species that belong to the bacterial order Clostridiales, particularly members of the Clostridium clusters XIVa and IV [43–45]. One member of this Clostridiales order that is drastically reduced in the ileum of patients with CD is *Faecalibacterium prausnitzii* [46].

Since Nod2 is an intracellular microbial sensor for gram-positive and gram-negative bacteria, it has been proposed that *Nod2* deficiency or mutations can contribute to the modification of microbial composition, and then disease development. In humans, an increased load of Bacteriodetes was observed in the ileal mucosa of CD patients with homozygosity in *NOD2* mutations [47]. *NOD2* mutations have also been associated with an increased load of *Escherichia coli* (Proteobacteria) and a reduced load of *F*. *prausnitzii* (Firmicutes) [45,47–49]. In mice, numerous studies have reported the key role played by Nod2 in the maintenance of the gut microbiota [21,47,50–55]. Compared to control mice, *Nod2*^KO^ mice display an increased frequency of the Bacteriodetes phylum and a decrease in the Firmicutes phylum in intestine and feces [21,47,50–55]. As the modifications of the microbiota linked to *Nod2* deficiency at genus level is dependent on the conditions of animal housing, the identification of bacterial species impacted by Nod2 remains difficult to establish. Although microbial dysbiosis in *Nod2*^KO^ mice have been reported by several groups, two studies failed to show significant differences in the gut microbiota when *Nod2*^KO^ and wild-type (WT) mice were cohoused [56,57]. Indeed, if the cage effect, drift in independent lines, coprophagia, and genetic background have not all been taken into consideration, studies investigating microbiota communities in genetically altered mice are often misleading. Cohousing seems to be a very rigorous strategy, but the absence of any difference between WT and *Nod2*^KO^ mice [56,57] may result from coprophagia and the subsequent homogenization of mouse microbiota [57]. Indeed, *Nod2*^KO^ mice obtained by embryo transfer into WT mice exhibit an intestinal microbiota different from their mothers but similar to that of single-housed *Nod2*^KO^ mice [53]. Thus, the use of embryo transfer strategy, which reduces the impact of environmental and mother parameters, points out the role of *Nod2* deficiency in the active acquisition of dysbiosis [53]. WT and *Nod2*^KO^ mice obtained by embryo transfer into WT mother mice exhibit the same microbiota when housed in the same cage, confirming the homogenization of the gut microbiota between cohoused mice (likely through coprophagia). Moreover, the difference in intestinal flora between WT and *Nod2*^KO^ offspring and their WT mothers shows that microbial dysbiosis linked to *Nod2* deletion is transmissible and dominant [53]. Moreover, microbiota dysbiosis, which occurs in *Nod2*^KO^ but also in *RIP2*^KO^ mice, may enhance sensitivity to both colitis and colonic adenocarcinoma. Sensitivity to colitis is transmissible to WT mice via the microbiota after cohousing. Since diet dominates host genotype in shaping the gut microbiota [58], a common dysbiosis shared by people in close contact might explain development of CD in spouses of CD patients and the nonrandom distribution of CD within multiplex sibships [59].

The mechanisms by which *Nod2* regulates microbiota communities in the gut are still unclear, even though it is commonly admitted that Nod2 in intestinal epithelial cells plays a major role by promoting the production of antibacterial compounds, including defensins, by Paneth cells [19,29,48,54,60–62]. The impact of the genetic background in the effect of Nod2 deficiency on the expression of defensins is, however, matter of debate [29,57]. Goblet cell abnormalities, including decrease in number and mucins secretion [21], have also been reported to be linked to *Nod2* deficiency. The failure in goblet cell function was associated with an overproduction of IFN-γ by intraepithelial lymphocytes and the expansion of *Bacteroides vulgatus*.

Nod2 not only regulates the bacterial load and microbiota composition but also plays a key role in shaping bacterial translocation and attachment on gut epithelium. Indeed, *Nod2*^KO^ mice exhibit an increased bacterial translocation of both gram-positive and gram-negative bacteria and the yeast *Saccharomyces cerevisiae*. This barrier defect is specifically located at Peyer’s patches in the ileum [63]. Although commensal *E*. *coli* may attach at all intestinal segments [64], adherent-invasive *E*. *coli* (AIEC), known to be associated with CD, has an excessive capacity to attach at the surface of Peyer’s patches in *Nod2*^KO^ mice [65]. The infiltration of T helper type 1 (Th1) lymphocytes (secreting TNF-α and IFN-γ) resulting in an overexpression of the myosin light chain kinase (MLCK) in epithelial cells was proposed as a mechanism for bacteria translocation across the Peyer’s patches [66]. Similarly, a bacteria-induced overactivation of the MLCK may increase the number of TGF-β-producing regulatory CD4^+^ T cells in the colonic lamina propria of *Nod2*^KO^ mice through the induction of an excessive permeability [67]. This reciprocal link between immune cells, intestinal permeability, and microbiota is further evidenced by the fact that endocytosis of commensal bacteria in epithelial cells is dependent on MLCK-activated brush border fanning triggered by IFNγ [68,69]. Thus, Nod2, by regulating the load and the composition of the microbiota, the passage of the intestinal barrier, and the immune response against the intestinal flora (including innate but also Th1, Th2, and Th17 adaptive immunity), acts as a primordial barrier guard [70–73].

## NOD2 and pathogens

In addition to its role in the regulation of gut microbiota in normal conditions, NOD2 is involved in the host response against infectious pathogens, including bacteria, viruses, and parasites. A large literature reported that TLR stimulation, required to initiate innate and adaptive immunity upon infection, is modulated by NOD2 [74]. However, as pathogens are sensed by multiple PRRs, *Nod2* deficiency has only modest effects on pathogen clearance in vivo [75]. In addition, as exemplified in *Brucella abortus* infection, Nod2 may also induce inflammation via endoplasmic reticulum stress/Nod2/RIP2 pathway [34].

## Bacteria

Since NOD2 is expressed in hematopoietic and nonhematopoietic cells and is able to recognize a fragment of PGN from gram-positive and gram-negative bacteria, it is involved in the control of a large panel of pathogenic bacteria. Over the last 10 years, Nod2 has emerged as a key player in the control of pathogenic bacteria like *Campylobacter*, *Citrobacter*, *Escherichia*, *Helicobacter*, *Listeria*, *Mycobacteria*, *Pseudomonas*, *Staphylococcus*, *Yersinia*, and other species. The variety of the cellular and animal models, as well as the large spectrum of bacterial strains, has led to the identification of many signaling pathways involving Nod2, which sometimes may be contradictory for the same pathogenic bacteria genus. However, the recruitment of RIP2/TAK1 complexes by Nod2 is consistently required to control bacterial infection and related inflammation (Table 1).

**Table 1:** Role of Nod2 in the host response toward pathogenic bacteria.

Bacteria	Bacterial susceptibility in *Nod2*^*KO*^	Intestinal inflammation in *Nod2*^*KO*^	Cytokines/ chemokines in *Nod2*^*KO*^	Intestinal permeability	Nod2 and TLR synergy	RIP2 mediated	Activation of Caspase-1 and IL-1β	Refs
*Yersinia pseudotuberculosis*, *Y*. *enterocolitica*	Decreased	Exacerbated	IL-1β decreased	Increased in WT mice. Unchanged in *Nod2*^*KO*^ mice.	Yes (TLR2)	Yes	Yes	[39,87,88]

*Listeria monocytogenes*	Increased	Not studied	IL-6, IL-12 & TNF-α decreased	Increased in WT mice. Not studied in *Nod2*^*KO*^ mice.	Not studied	Yes	Not studied	[29,98]
*Pseudomonas fluorescens*	Decreased	Absent	IL-1β & TNF-α decreased	Increased in WT mice. Unchanged in *Nod2*^*KO*^ mice.	Not studied	Yes	Yes	[105,108]
*E*. *coli*	Increased (attachment to M-cells enhanced)	Absent	TNF-α decreased	Increased in WT mice.	Not studied	Not studied	Not studied	[65,122]
*Citrobacter rodentium*	Increased	Reduced at day 12 Increased at day 22	IFN-γ, IL-17 α CCL2 decreased	Not studied	Not studied	Yes	Not studied	[123, 124, 125]
*Campylobacter jejuni*	Unchanged in *Nod2*^*KO*^ mice. Increased in *IL10/Nod2*^*KO*^ mice	Absent in *Nod2*^*KO*^ mice. Exacerbated in *IL10/Nod2*^*KO*^ mice.	Unchanged in *Nod2*^*KO*^ mice. IL-1β, TNF-α & CxCL1 decreased in *IL10/Nod2*^*KO*^ mice.	Not studied	Not studied	Not studied	Yes	[135,136]
*Heliobacter hepaticus*	Increased	Exacerbated	IFN-γ increased	Not studied	Not studied	Yes	Not studied	[72,110]

### Yersinia

*Yersinia* genus, a gram-negative rod-shaped bacteria, contains about ten species. Three species are pathogenic for humans and rodents: *Y*. *enterocolitica*, *Y*. *pestis*, and *Y*. *pseudotuberculosis*. *Y*. *enterocolitica* and *Y*. *pseudotuberculosis* are enteropathogens, able to invade the host through Peyer’s patches [76–78]. *Y*. *pestis* is the causative agent of the systemic invasive infectious disease known as plague [79]. All of them cause a wide range of symptoms and pathologies, including diarrhea, gastroenteritis, and mesenteric adenolymphitis, in both humans and rodents [80,81]. These infections are usually acquired by ingestion of contaminated food or water. In mice, oral inoculation with enteropathogenic *Yersinia* results in translocation of bacteria from the intestine to the spleen and liver and leads to animal death [82]. In some cases, especially in patients with a compromised immune system, enteric *Yersinia* may disseminate systemically [83,84].

Initial reports on humans suggested that Nod2 is involved in the recognition of pathogenic *Yersinia* species [85,86]. Peripheral blood mononuclear cells (PBMCs) from homozygous carriers of the *NOD2*^*3020insC*^ mutation display lower production of anti-inflammatory cytokines in response to *Y*. *enterocolitica*, *Y*. *pestis*, or *Y*. *pseudotuberculosis* [85]. IL-6 production induced by *Y*. *enterocolitica* was also impaired in PBMCs from a patient with *NOD2* mutations and chronic yersiniosis [86]. When orally inoculated, *Y*. *pseudotuberculosis* induces an ileal inflammation associated with an altered permeability of the intestinal barrier mediated by TLR2 [87] and Nod2 signaling [39,88]. *Yersinia* virulent factor YopJ exacerbates this effect by blocking the NOD2/RIP2/TAK1 signaling pathway and thus facilitating Nod2/Caspase-1 interaction with a subsequent production of IL-1β. In case of *Nod2* deficiency, YopJ is no more able to activate the Nod2-dependant Caspase-1 signaling pathway, limiting the ileal inflammation at the beginning of enteral infection [39]. This effect is sufficient to reduce the mortality rate of *Nod2*^KO^ mice orally inoculated with *Y*. *pseudotuberculosis*. By contrast, in naive bone-marrow-derived macrophages (BMDMs), NOD2 [89] and RIP2 [90,91] are dispensable for innate immune response against *Y*. *enterocolitica*. The production of cytokines and nitric oxide, the activation of NF-κB and MAPK, and the phagocytic activity remain unchanged in *Yersinia*-infected BMDMs from *Nod2*^KO^ mice [91]. In agreement, Meinzer et al. showed that Nod2 was critical in case of infection by *Y*. *pseudotuberculosis* via the oral (but not systemic) route in mice [88].

### Listeria monocytogenes

*Listeria monocytogenes* is a causative agent for human listeriosis, a potentially fatal foodborne infection. *L*. *monocytogenes* is an intracellular pathogen phagocytosed by monocytes/macrophages that escape from the phagosome into the host cell cytosol via its pore-forming toxin listeriolysin O (LLO) [92]. *L*. *monocytogenes* also invades nonphagocytic cells, such as enterocytes and M cells. This process is critical for bacterial translocation through the intestinal epithelium [93–95]. The role of Nod2 in the response against *L*. *monocytogenes* is controversial. In an earlier study, Kobayashi and collaborators reported that *Nod2*^KO^ mice challenged with *L*. *monocytogenes* via the intragastrical route are more susceptible to infection, with higher translocation rates from the intestine to the liver and spleen [29,96]. This phenotype is lost in the case of systemic infection. In a later study, *Rip2*^KO^ mice were shown to be highly susceptible to systemic *Listeria* infection [97]. In infected *Nod2*^KO^ mice, the number of *L*. *monocytogenes* was not increased in Peyer's patches, suggesting an M cell-independent route of bacterial invasion [29]. To explain the hypersensitivity to *Listeria* infection, the authors reported a decrease in the production of defensin-related cryptdin 4 (Defcr4) and Defcr-related sequence 10 (Defcr-rs10) by Paneth cells in *Nod2*^KO^ mice [29]. However, the Sartor group recently reported that WT and *Nod2*^KO^ mice produced similar levels of a large number of cryptins/α-defensins but do not express Defcr4 [57].

Contradictory results about the role of *NOD2* in the induction of pro-inflammatory cytokines by macrophages in response to infection by *L*. *monocytogenes* were also reported in vitro [98,99]. RNA interference and other Nod2 inhibition experiments in human PBMCs, as well as experiments using BMDMs from NLRP3 or RIP2^KO^ mice, demonstrated that *Listeria*-induced IL-1β release was dependent on apoptosis-associated speck-like protein containing a CARD (ASC), Caspase-1, and NLRP3, whereas NOD2, RIP2, NLRP1, NLRP6, NLRP12, NLRC4, and absent in melanoma 2 (AIM2) appeared to be dispensable [100]. Furthermore, in murine BMDMs, Nod1 and Nod2 seem to have redundant functions with regards to *Listeria* infection. Nod1 or Nod2 deficiency alone does not result in a significant alteration in cytokine response to *Listeria* infection, while cytokine production is downregulated in *Rip2*^KO^ and *Nod1-Nod2*^DKO^ macrophages [101]. Attachment of bacteria to the cell surface is sufficient to activate macrophages [102]. This finding is consistent with the observation that Nod2 and RIP2 cooperate with TLR signaling for optimal responses to TLR ligands [101].

### P. fluorescens

*P*. *fluorescens* is present at low numbers in the intestinal lumen and in many ecological niches, including soil, water, and refrigerated food [103]. Although *P*. *fluorescens* has long been considered a psychotrophic microorganism, some clinical strains have been able to adapt at a growth temperature of 37 °C [104]. Clinical strains of *P*. *fluorescens* were shown to increase the paracellular permeability, cell cytotoxicity, and cytokine response in human enterocyte cells lines [105–107]. In vivo, *P*. *fluorescens* increases the paracellular permeability of the intestinal mucosa via the release of IL-1β by immune cells and the activation of MLCK in the epithelial cells in a Nod2-dependent way [108].

### H. hepaticus

*H*. *hepaticus* is the best studied member of the enterohepatic *Helicobacter* species. This gram-negative microaerophilic bacterium is an opportunistic pathogen [109] that induces colitis in immunodeficient mice. In both *Nod2*^KO^ and *Rip2*^KO^ mice, *Helicobacter* has been associated with the development of colitis (resembling human IBD) and cancer [110]. *Nod2*^KO^ and *Rip2*^KO^ mice were reported to be unable to regulate the *H*. *hepaticus* load in ileum [72]. Both of them develop a granulomatous ileitis and enlarged Peyer’s patches and mesenteric lymph nodes, with an expansion of IFNγ-producing CD4 and CD8 T cells [72]. Inflammatory Th1 response is associated with Nod2 expression in the crypts of the small intestine, suggesting a role for Paneth cells [72].

### Mycobacteria

*Mycobacteria* are an important group of pathological microorganisms. Worldwide, 2,000,000,000 people are infected with *M*. *tuberculosis*, and 2 million people die from tuberculosis each year [111]. Other mycobacterial species, such as *M*. *leprae*, are endemic in developing countries and are responsible for high morbidity and disability rates [112]. In patients with a compromised immune system, nonpathogenic mycobacteria may also cause disease. *M*. *avium paratuberculosis* (MAP) has been suggested to be associated with CD. This suggestion is controversial, but some findings support a causative role of MAP in the pathogenesis of CD [113]. In cattle, MAP causes Johne disease, which clinically resembles CD [114]. Furthermore, MAP has been identified by PCR and sometimes by culture in gut biopsies from CD patients [115].

As *M*. *paratuberculosis* and *NOD2* have been involved in CD, the role of NOD2 in the regulation of host susceptibility to *M*. *paratuberculosis* has been investigated [116]. NF-κB activation in NOD2-transfected HEK293 cells was found to be dose-dependent on MAP exposure [116]. Moreover, MAP-infected PBMCs from CD patients synthetize less inflammatory cytokines in case of *NOD2* mutations [116]. Of note, genomewide association studies have evidenced an association between *NOD2* and *RIP2* polymorphisms and leprosy caused by *M*. *leprae* [117]. Recently, synthesis of characteristic *Mycobacterium* PGN fragments has been shown to modulate the innate immune responses of Nod1 and Nod2 [118].

### E. coli

*E*. *coli* is widely spread in many ecological systems, including the human gut, where most bacteria are friendly commensal but a few strains are well-known pathogens [119]. Pathogenic *E*. *coli* strains are divided into two major groups: extra-intestinal pathogenic *E*. *coli* (ExPEC) and intestinal pathogenic *E*. *coli* (InPEC). Among the InPEC strains causing diarrheagenic infections, several well-defined pathotypes have been identified, including enteropathogenic *E*. *coli* (EPEC), enterotoxigenic *E*. *coli* (ETEC), enterohemorrhagic *E*. *coli* (EHEC), enteroaggregative *E*. *coli* (EAEC), enteroinvasive *E*. *coli* (EIEC), and AIEC [119]. AIEC interact with mouse and human Peyer’s patches via long polar fimbriae (LPF) and translocate across the M cells at the surface of Peyer’s patches[65]. AIEC are abnormally present in chronic ileal lesions of CD [120,121], and they frequently exhibit the LPF operon [65]. Although *Nod2*^KO^ mice do not develop macroscopic lesions of colitis, gut colonization by AIEC does not require antibiotics as for WT mice [65,122].

### C. rodentium

*C*. *rodentium* is a mouse-restricted pathogen. It colonizes intestinal mucosa and shares several pathogenic mechanisms with EPEC and EHEC, which are two clinically important human gastrointestinal pathogens [123]. *C*. *rodentium* induces a marked infiltration of inflammatory cells ten days after infection, and the colonization is resolved three weeks later [124]. The development of a humoral response against *C*. *rodentium* is required for this clearance [125]. Nod2 regulates the bacterial clearance by controlling the production of CCL2 and the subsequent influx of circulating inflammatory monocytes at the site of infection [126]. The regulation of CCL2 by Nod2 is mediated by hematopoietic and nonhematopoietic cells [126]. Colonic stromal cells producing CCL2 and pro-inflammatory CCR2-expressing Ly6C^hi^ monocytes are required for the clearance of *C*. *rodentium* [126]. Signaling pathways involved in Nod2-mediated clearance of *C*. *rodentium* include activation of NF-κB, MAPKs, and inflammasome, as well as autophagy [127–130].

### C. jejuni

*C*. *jejuni* is a gram-negative spiral-shaped bacteria that colonizes and survives as a commensal in the gastrointestinal tract of many animals and humans [128]. It is the foremost cause of bacterial foodborne diarrheal diseases worldwide, with up to 2.4 million cases annually in the United States alone. The main sources of transmission to humans are the consumption and handling of contaminated poultry. The “invasive” nature of *C*. *jejuni* led to investigation of the contribution of cytoplasmic PRRs as Nod1 and Nod2 in initiating the host response. Zilbauer et al. suggested that NOD1 (but not NOD2) is a potential PRR for *C*. *jejuni* in intestinal epithelial cells in vitro [131]. In agreement, although *C*. *jejuni* products elicit an inflammatory response from intestinal epithelial cells through the activation of NF-κB and the release of CXCL8 [132,133], *Nod2*^KO^ mice failed to develop colitis [134,135]. However, NOD2 signaling seems critical to control campylobacteriosis in *IL-*10^*KO*^ mice by improving nitric-oxide-dependent bactericidal activity [135].

## Viruses

During infection with viruses, TLR activation induces the production of type I IFN, which plays an important role in antiviral defense [136,137]. TLR-recognizing viral motifs include TLR3 for viral double stranded RNA [138], TLR7 and TLR8 for viral single stranded RNA [139], TLR9 for DNA containing unmethylated CpG motifs present in numerous viral pathogens, and TLR13 for bacterial ribosomal RNA. The regulatory role of Nod2 in viral infections is related to its capacity to sense microbiota-derived MDP and to modulate the TLR pathways activated by RNA and DNA viruses, including respiratory syncytial virus (RSV), influenza A virus (IAV), human immunodeficiency virus type-1 (HIV-1), norovirus (NV), and human enterovirus species B (HEV-B) (Table 2). MDP upregulates the production of IFN-β in PBMCs infected by RSV [140], a response that is lost when NOD2 is mutated [140]. In agreement with the role of Nod2 in antiviral response, *Nod2*^KO^ and *RIP2*^KO^ mice are hypersensitive to infection with RSV. This hypersensitivity is associated with a failure in mitochondria autophagy and superoxide overproduction, resulting in mitochondrial damage and activation of the NLRP3 inflammasome and subsequent IL-18 release [141]. Nod2 also regulates the innate anti-RSV response via its interaction with the adaptor protein MAVS (mitochondrial antiviral signaling) [142].

**Table 2:** Role of Nod2 in the host response toward viruses.

Bacteria	Viral susceptibility in *Nod2*^*KO*^	Nod2 expression	Viruses replication/ reactivation *Nod2*^*KO*^	Viral clearance in *Nod2*^*ko*^	Nod2 and TLR synergy	Enhanced inflammatory cytokines/ chemokines	RIP2 mediated	Refs
RNA viruses								
RSV	Increased	Enhanced	Reduced	Enhanced	Yes (TLR3)	IL-1β & TNF-α	Yes	[141,142]
IAV	Increased	Not studied	Reduced	Enhanced	Not studied	IFN-γ	Not studied	[143]
HIV-1	Not studied	Enhanced	Enhanced		Not studied	CXCL8	Not studied	[144]
NV	Decreased	Enhanced	Not studied	Not studied	Yes	TNF-α	Yes	[145]
RNA viruses								
Human cytomegalovirus (HCMV)	Not studied	Yes	Increased replication	Not studied	Not studied	CXCL8	Yes	[146]
Human herpes viruses (HVs)	Not studied	No	Increased reactivation in case of *NOD2* mutation	Not studied	Not studied	Not studied	Not studied	[147]

Although the innate immune system is able to trigger an inflammatory response to viruses, efficient clearance requires the combined efforts of both innate and adaptive immunity. Indeed, *Nod2*^KO^ mice infected IAV exhibit reduced IFN-β levels, fewer activated dendritic cells, and virus-specific CD8^+^ T cells that produce low levels of IFN-γ. *Nod2*^KO^ dendritic cells have a lower costimulatory capacity and are more prone to cell death [143]. Similarly, some RNA viruses, such as HIV-1, may impact adaptive T cell response via the activation of dectin-1/TLR2 and NOD2 in dendritic cells [144]. Moreover, infection by RNA viruses, including RSV, NV, and HIV-1, is commonly associated with Nod2 upregulation, which results in the overproduction of TNF-α [145].

Nod2 is also involved in the control of the replication or reactivation of DNA viruses, including HCMV and HVs. Similar to RNA viruses, HCMV upregulates *NOD2* as early as two hours post-infection and for up to 24 hours afterward [146]. As shown in HCMV-infected cells, the overexpression of *NOD2* or its downstream kinase RIP2 leads to the production of both IFN-β and pro-inflammatory cytokines/chemokines [146]. Conversely, *NOD2* deficiency, as well as *NOD2*^3020insC^ mutation, downregulates both IFN-β and CXCL8, thereby favoring HCMV replication [146]. In contrast to HCMV, HV is not able to upregulate NOD2. However, the NOD2 mutation SNP8 (2104C>T) has been associated with HV reactivation and bacteremia, with both occurring after allogeneic hematopoietic stem cell transplantation [147].

## Parasites and yeasts

Little is known about the role of NOD2 in parasitic or fungal infections. Over the last ten years, growing evidence has reported that Nod2 could be instrumental in controlling *Toxoplasma gondii* infection, while its role in *Leishmania*, *Trypanosoma cruzia*, and *Candida albicans* infections remains minor (Table 3). *T*. *gondii* is an obligate intracellular protozoan pathogen able to infect various animal species, leading to severe diseases, including pneumonia and encephalitis, in immunocompromised hosts. The outcome of *T*. *gondii* infection is dependent on the ability of the host to elicit a robust cellular immune response, particularly the production of IFN-γ by natural killer cells and Th1 lymphocytes [148]. The role of Nod2 in the protection of the host is supported by the demonstration that the administration of *T*. *gondii* orally induces a more severe ileitis in *Nod2*^KO^ mice than in WT mice [149]. Infected *Nod2*^KO^ mice display an increase in the parasitic load in the small intestine and the brain and a higher translocation of bacteria from the gut to the liver, spleen, and kidneys [149]. Reconstitution of T cell-deficient mice with *Nod2*^KO^ T cells followed by *T*. *gondii* infection demonstrated an intrinsic defect of *Nod2*^KO^ T lymphocytes to produce IL-2 and differentiate into Th1 lymphocytes [14]. Based on an inverse correlation between *Nod2* transcript levels and the intracellular survival of *Leishmania infantum* in macrophages [150], it has been proposed that Nod2 might also play a role in host defense against *Leishmania*. By contrast, Nod2 has virtually no impact on the outcome of the infections with *T*. *cruzi* [151] and *C*. *albicans* [152], although chitin particles from the commensal yeast *C*. *albicans* induce IL-10 through Nod2 and TLR9 pathways [153]. Finally, a positive association between NOD2 mutations linked to CD and elevated levels of anti-*Saccharomyces cerevisiae* antibodies in the serum of CD patients has been described [154].

**Table 3:** Role of Nod2 in the host response toward parasites and yeasts.

Parasites/yeasts	Intestinal inflammation in *Nod2*^*KO*^	Association between *NOD2* mutations and parasite infection	Parasitic load in *Nod2*^*KO*^	Cytokines in *Nod2*^*KO*^	Refs
*T*. *gondii*	Exacerbated	Not studied	Increased	IFNγ & IL-12 decreased	[14,148, 149]
*Leishmania* spp.	Not studied	Not studied	Increased	Not studied	[150]
*T*. *cruzi*	Not studied	Not studied	Unchanged	Unchanged	[151]
*C*. *albicans*	Not studied	None	Not studied	Unchanged or IL-10 increased	[152, 153, 154]

## Concluding remarks

The mucosal surfaces of the intestinal tract are constantly exposed to complex microbial communities containing commensal microorganisms and sometimes pathogens. Hosts harbor multiple mechanisms to maintain intestinal barrier integrity and immune tolerance toward commensal bacteria while reacting against pathogens. In this context, NOD2 plays a key role in gut–microbe homeostasis by sensing both commensal and pathogenic microbes and modulating TLR signaling pathways.

CD and UC result from a chronic, uncontrolled immune response against components of the intestinal microbiome in genetically susceptible hosts. Initiation and/or relapse of IBDs are often associated with pathogenic microbes, including bacteria, viruses, and parasites. In genetically predisposed individuals, IBDs occur due to an alteration of the subtle interplay between resident microbiota and the immune system, which often originates from intestinal barrier dysfunction. Over the last 20 years, a large number of studies reported that pathogens (such as *Y*. *pseudotuberculosis*, *Y*. *enterocolitica*, *P*. *fluorescence*, AEIC, and *L*. *monocytogenes*) and/or an altered microbiota are often involved in the physiopathology of IBDs. All these bacterial strains may alter paracellular permeability and favor bacterial translocation, but their detrimental effects on host intestinal mucosa are downmodulated by Nod2. CD has also been associated with CMV infection and, to a lesser degree, HV, rotavirus, NV, and adenovirus, all of which alter intestinal permeability. Replication and/or reactivation of most of these viruses, as well as the cycles of parasites and/or yeasts (known to alter intestinal permeability), are regulated by NOD2. Furthermore, *Nod2* deficiency is often associated with exacerbated immune responses against pathogens as diverse as bacteria (*Y*. *pseudotuberculosis*, *H*. *hepaticus*), parasites (*T gondii*), and viruses (Norovirus). Although the regulatory role of *NOD2* in the response of the host against pathogens is largely admitted, its impact on microbiota composition is still a matter of debate. The main difficulty is controlling the environmental parameters known to influence microbiota composition, such as coprophagia, which homogenizes microbiota upon cohousing. The transfer of *Nod2*^KO^ embryos into WT mothers, which represents an experimental alternative to overcome misleading results, has shown that *Nod2* deficiency results in dominant and transmissible microbial dysbiosis. The mechanisms involved and the role of bacterial dysbiosis in the development and/or aggravation of IBD remain unclear, however. Indeed, *Nod2*^KO^ mice display high numbers of CD4^+^ T cells in Peyer’s patches and an increased intestinal permeability, but the transfer of microbiota from *Nod2*^KO^ mice to WT mice alters neither CD4^+^ T lymphocyte count nor permeability. By contrast, the decreased production of both antimicrobial peptides and mucins by nonhematopoietic cells, as well as susceptibility to colitis, may be acquired by transferring *Nod2*^KO^-associated dysbiosis. However, the impact of bacterial dysbiosis on pathogen implantation and vice versa, as well as the contribution of pathogens to the effects of dysbiosis on intestinal inflammation, still remains to be determined.
